# The *RAS* oncogene in brain tumors and the involvement of *let-7* microRNA

**DOI:** 10.1007/s11033-024-09439-z

**Published:** 2024-04-18

**Authors:** Samantha Messina

**Affiliations:** Department of Science, Roma Tre University, Viale Guglielmo Marconi 446, 00146 Rome, Italy

**Keywords:** *RAS* oncogenes, *Let-7* miRNA, Brain tumor

## Abstract

*RAS* oncogenes are master regulator genes in many cancers. In general, *RAS*-driven cancers have an oncogenic *RAS* mutation that promotes disease progression (colon, lung, pancreas). In contrast, brain tumors are not necessarily *RAS*-driven cancers because *RAS* mutations are rarely observed. In particular, glioblastomas (the most lethal brain tumor) do not appear to have dominant genetic mutations that are suitable for targeted therapy. Standard treatment for most brain tumors continues to focus on maximal surgical resection, radiotherapy and chemotherapy. Yet the convergence of genomic aberrations such as EGFR, PDGFR and NF1 (some of which are clinically effective) with activation of the *RAS*/MAPK cascade is still considered a key point in gliomagenesis, and *KRAS* is undoubtedly a driving gene in gliomagenesis in mice. In cancer, microRNAs (miRNA) are small, non-coding RNAs that regulate carcinogenesis. However, the functional consequences of aberrant miRNA expression in cancer are still poorly understood. *let-7* encodes an intergenic miRNA that is classified as a tumour suppressor, at least in lung cancer. *Let-7* suppresses a plethora of oncogenes such as *RAS*, HMGA, c-Myc, cyclin-D and thus suppresses cancer development, differentiation and progression. *let-7* family members are direct regulators of certain *RAS* family genes by binding to the sequences in their 3′untranslated region (3′UTR). *let-7* miRNA is involved in the malignant behaviour in vitro—proliferation, migration and invasion—of gliomas and stem-like glioma cells as well as in vivo models of glioblastoma multiforme (GBM) via *KRAS* inhibition. It also increases resistance to certain chemotherapeutic agents and radiotherapy in GBM. Although *let-7* therapy is not yet established, this review updates the current state of knowledge on the contribution of miRNA *let-7* in interaction with *KRAS* to the oncogenesis of brain tumours.

## Introduction

MicroRNAs (miRNAs) are a class of non-coding RNAs that function as endogenous triggers of the RNA interference pathway. In general, aberrant expression of microRNAs has been linked to all human cancers. miRNAs are dysregulated in a plethora of diseases, including cancers; poorly differentiated tumors displayed lower miRNA expression compared to tumors with a higher differentiation. The Slack laboratory showed one of the first pieces of evidence that *lethal-7 (let-7)* plays a key role in cancer. By showing that *RAS* is regulated by the *let-7* miRNA family in *Caenorhabditis elegans* and by highlighting *let-7* complementary sites in human *RAS* 3′UTRs, they first validated that the *let-7* miRNA family negatively regulates *RAS* in two different *C. elegans* tissues and human cell lines and lung tissue. This was the first report of the mechanism of action of a miRNA acting as a tumor suppressor [1, 2].

The heterochronic miRNA *let-7* display tumor suppressive properties at least in lung, colon, breast, and leukemia cancers. It is categorized as a tumor suppressor because it reduces cancer aggressiveness, chemoresistance, and radio-resistance. Particularly, studies inferred from human cancer databases report *let-7* family member expression associated with poor overall survival in some cancers; but regretfully with unsignificant results in glioblastoma multiforme (GBM) [3].

By contrast, many in vitro studies report that *let-7* inhibits the malignant behavior of glioma cells and stem-like cells [4–13] (Table 1). Given the promiscuous and context-specific nature of miRNA targeting, many mechanisms of interactions remain to be elucidated. Moreover, regulation of *RAS* protein level and *RAS*/MAPK cascade are regulated by a myriad of miRNAs family without a clear mechanistic link [11, 12, 14–16]. Three recent reports focused on miRNAs targeting *RAS* in GBM and showed that miR-143-3p directly targets *NRAS* [17], let-7a-5p directly targets *KRAS* [7], and both R-Ras and N-Ras (related *RAS* viral oncogene homolog, *HRAS* homolog) are direct targets of miR-124-3p [18].

**Table 1:** *let-7* target genes in glioma cancer

*Let 7* target gene	Cancer brain type	Phenotype	Experimental system	References
TLR7	Glioma	–	In vitro	Buonfiglioli et al. [8]
–	**Glioblastoma**	**DNA-repair**	**In vitro**	**Chaudhry et al. **[19]
IMP2	GSC	–	In vitro	Degrauwe et al. [4]
–	GSC	GSC/GBM transition	In vitro	Evers et al. [20]
Cyclin D1	Glioblastoma	Response to chemotherapy	In vitro	Guo et al. [21]
K-Ras	Glioma	Proliferation	In vitro	Lee et al. [10]
HMG2A	Glioma	Proliferation and invasion	In vitro	Li et al. [5]
–	**Human neural stem cell**	**Tumorigenesis and invasion**	–	**Mao et al. **[22]
E2F	Glioblastoma	Malignant behavior	In vitro	Song et al. [6]
K-Ras	Glioma	Malignancy	In vitro and in vivo	Wang et al. [7]
MDM4	Glioma	DNA damage	In vitro	Xie et al. [13]

Target genes of *let-7* in preclinical models of glioma are listed in the table with cancer type and experimental model specifications. Bold references indicate that target gene characterization is lacking. This list focuses on aspects relevant to the current paper and is not intended to be truly comprehensive

*let-7* is a *bona fide* tumor suppressor gene, but this categorization is rarely straightforward, since some miRNA can have both oncogenic and tumor-suppressive mechanisms, depending on the context. *Let-7* targets multiple oncogenes (*RAS*, c-MYC, HMGA2 to date) and the prominent mechanisms by which *let-7* exerts a tumor suppressive role is by repressing the translation of the three *RAS* proteins (*HRAS*, *NRAS*, and *KRAS*) and c-MYC, a downstream effector of RAS-ERK signaling [1, 23–27]. Moreover, oncogenic functions for *let-7* have been also reported in some cases [28, 29]. Indeed, the functions of *let-7* have been reported not only in cancer but also in other diseases, including viral infection, immune diseases, neurological diseases, diabetes, and cardiovascular diseases. In vivo studies with Cre-inducible *let-7-*transgenic mice have reported a strong phenotypic read-out both in diabetic retinopathy and in diabetes [30, 31].

As a proto-oncogene that regulates several oncogenic pathways, *KRAS* has always been considered a central signaling modulator. Thus, there is a flourishing literature on studying miRNAs that regulate K-RAS expression, with *let-7* being of prime importance. Our knowledge of how miRNAs can modulate activation of the RAS-ERK signalling pathway continues to grow as potential miRNA-mRNA regulatory networks are identified using different strategies [32]. From these studies, three main paradigms of miRNA-mediated RAS-ERK regulation have emerged. miRNAs can affect the translation of (i) core components of the RAS-ERK pathway (e.g., *let-7* targets *HRAS*, *NRAS* and *KRAS*) [1, 33], (ii) critical proteins that regulate the pathway and are required for proper spatial and temporal control of RAS-ERK signalling [29, 34, 35], and (iii) upstream drivers and downstream effector/regulator molecules [36, 37]. A myriad of miRNAs regulates RAS-ERK pathway activity in a variety of cancer contexts [38, 39].

In general, there is a very clear correlation between the loss of *let-7* expression and the development of poorly differentiated and aggressive cancers, this is the case for *let-7a* in lung carcinomas. In prototypic studies in non-small cell lung cancer (NSCLC) models, *let-7* expression was analysed in vitro and in vivo and its role in KRAS-mediated NSCLC tumorigenesis was demonstrated [40]. *let-7* inhibits tumour development and the RAS-ERK signalling pathway in an autochthonous model of NSCLC driven by activated *KRAS* (KRASG12D) [41, 42]. In xenograft models of NSCLC, *let-7* exerts a tumour suppressive role [24] and increased expression of *let-7a* significantly reduces tumour burden in a K-Ras lung cancer model in mice [43]. Furthermore, *let-7* counteracts the maintenance, survival and self-renewal of cancer stem-like cells (CSCs) in ovarian and breast cancer, and this suppressive activity correlated with reduced expression of *RAS* and HMGA2 [43, 45, 46, 47]. Thus, by suppressing RAS expression, *let-7* can attenuate RAF-MEK-ERK signalling and its dependent oncogenic phenotypes independent of *RAS* mutation status. Functional studies have confirmed that miRNA *let-7* dysregulation is causative in many cancer cases, highlighting its potential impact on RAS-ERK signalling from a mechanism perspective.

## Main text

### *Let-7* expression in brain tumors

Brain tumours comprise a broad spectrum of over 120 histologically, demographically, clinically and molecularly different diseases (overview in [48]). Glioblastoma is the most common malignant primary brain tumour in adults and remains incurable [49]. Standard treatment for most brain tumours remains focused on maximal surgical resection, radiotherapy and chemotherapy with temozolomide (TMZ) as first-line therapy. Indirect targeting of the tumour by anti-angiogenics (e.g. bevacizumab) and immunotherapies (vaccines, adoptive therapies, immune checkpoint inhibitors and oncolytic viruses) have shown mixed efficacy (or inactivity) in preclinical studies [50,48, 51, 52, 53, 54]. Alternative studies focusing on molecular profiling of GBM identified neurofibromin-1 (NF1) as targeting mutations that contribute to activated *KRAS* signalling [55, 56].

The members of the *let-7* family are direct and strong regulators of K-RAS, N-RAS and H-RAS mRNAs through their 3′UTR sequences [15, 57, 58]. A single miRNA can control entire cellular signalling pathways by interacting with a broad spectrum of target genes. For example, *let-7* inhibits GBM tumour growth by interacting with a broad spectrum of target genes such as Ras, c-Myc, Stat3, Cyclin D1 [4, 6, 7, 10, 22] (see Table 1). Mature *let-7* can be blocked by the LIN28 protein, increased levels of which are associated with poorer survival in gliomas, and *let-7b* can serve as a marker for chemoresistance [20, 21]. In irradiated human glioblastoma cells, *let-7* mediated resistance to radiotherapy by regulating its relative expression [19]. Remarkably, the resulting loss of *let-7* enhances the expression of oncogenic targets such as *RAS* in loss-of-function and gain-of-function experiments [36].

The detection of miRNA has rapidly emerged as a potential biomarker in patients with glioblastoma [59, 60]. In particular, decreased *let-7b* has recently been associated with poor prognosis in gliomas [61]. Based on several high-throughput genomic technologies, The cancer genome atlas (TCGA) has defined RAS/MAPK as one of the central pathways involved in GBM [62] and *let-7* miRNA family expression levels are not reduced in GBM (summarised by [11, 12]). Regardless of its expression level, *let-7* miRNA can impair glioblastoma growth and cell migration through RAS inhibition [7, 10]. Specifically, forced expression of *let-7* miRNA reduced the expression of pan-RAS, N-RAS and K-RAS, thereby reducing proliferation and migration as well as tumour size in xenograft-transplanted GBM in nude mice [10]. In another study, overexpressed *let-7a* inhibited glioma cell malignancy by directly targeting *KRAS* independently of *PTEN* [7], and *let-7b* in turn inhibits malignant behaviour (proliferation, migration and invasion) of glioma cells and stem-like glioma cells [6]. Indeed, focal deletion of members of the *let-7* family (let-7a-2 and let-7e) has been found in medulloblastoma (MB) [63] and the *let-7* family has been validated in spontaneous and radiation-induced MB [64]. Conversely, miR-*let7g* was found to be upregulated in anaplastic and differentially expressed in desmoplastic MB [65, 66], although the functional consequences of its dysregulation have not yet been investigated. Recently, *let-7* miRNA activity has been identified as a prognostic biomarker of SHH medulloblastoma [67]. Furthermore, in a paediatric brain tumour (Diffuse intrinsic pontine gliomas, DIPG), *RAS* signalling has recently been identified as a novel therapeutic vulnerability and the RNA-binding protein LIN28B is overexpressed in DMG and suppresses the *let-7* family of microRNAs [68, 69]. Numerous oncogenes and signalling pathways besides *RAS* (e.g. MYC) have been shown to be targets of *let‐7* miRNAs, and KRAS is the target of many other miRNAs in gliomogenesis [5, 16]. Which of these targets genes determine the overall phenotype in glioblastoma remains to be investigated.

### *RAS* oncogenes in brain tumors

Comprehensive molecular profiling has dramatically changed the diagnostic neuropathology of brain tumours. Several of the key molecular alterations that are critical for glioma classification involve epigenetic dysregulation at a fundamental level and involve areas of biology not previously thought to play an important role in glioma pathogenesis [48]. The biological functions of the *RAS* family [the viral oncogene homologue of Harvey rat sarcoma (*HRAS*), the viral oncogene homologue of Kirsten rat sarcoma (*KRAS*) and the viral oncogene homologue of neuroblastoma (*NRAS*)] have been studied in detail for decades. Although only 1% of GBM tumours exhibit *RAS* mutation or amplification, 10% of GBM tumours contain inactivating genetic alterations of neurofibromin 1 (NF1) that lead to hyperactive RAS activity by enhancing intrinsic GTPase activity [62, 70]. Remarkably, dysfunctional signalling in tumours arises not only from gene mutations but also from epigenetic changes or pathway rewiring, which probably explains why there appear to be no dominant driver mutations in certain tumour types, most notably glioblastoma. Furthermore, no evidence of oncogenic mutations affecting *NRAS, KRAS, HRAS, BRAF* or *PDGFR* was found in medulloblastoma [71].

Deregulated *RAS* signalling is an important step in carcinogenesis, with activating *RAS* mutations playing a role in 30% of all cancers [72]. In contrast to many human tumours, *RAS* mutation is not common in human gliomas, with some exceptions such as cerebellar GBM [73]. Hyperactive *RAS* signalling alone is sufficient to generate gliomas that closely resemble human tumours in glioma mouse models. The *RAS* signalling pathway is therefore of central importance for human gliomagenesis. Primary GBMs are associated with impaired *RAS* signalling and expression of the oncogenic *HRAS* leads to a malignant phenotype in glioma cell lines [74, 75, 76, 77]. In particular, the *KRAS* oncogene is strongly involved in tumourigenesis in glioblastomas [75, 78, 79, 80], although *KRAS* mutations are almost absent in malignant gliomas [81]. Therefore, the observed deregulation of the Ras-RAF-ERK signalling pathway in gliomas is generally attributed to its upstream positive regulators, including EGFR and PDGFR, which are known to be highly active in many malignant gliomas [70]. It is likely that mechanisms other than mutations contribute to the activation of the RAS-MAPK signalling pathway in wild-type cancer. Indeed, epigenetic modifications have been described to enhance this activation in human tumours [82], and dysregulation of physiological miRNA activity has been shown to play an important role in gliomagenesis, but the functional significance of this regulatory level is currently unknown [11, 12, 75, 84, 85, 86, 87].

### Therapeutic and diagnostic potential of mirnas

Clinical studies on the benefits of miRNAs in diagnosis and therapy are already underway [88, 89]. MiRNAs are abundant non-coding RNAs, and their short length increases their stability compared to longer RNA molecules. In addition, miRNAs are secreted alone into the extracellular fluid or encapsulated by vesicles such as microvesicles and exosomes. Subsequently, the secreted miRNAs are found in the circulation. In summary, cancer-specific and circulating miRNAs are attractive diagnostic markers. Besides diagnostic miRNAs, miRNAs that can be used to predict drug efficacy and patient prognosis will greatly assist the advancement of precision cancer medicine. Despite the increasing interest in miRNA therapies as potential approaches for cancer treatment, the actual deployment of these molecules into solid tumors remains a formidable obstacle [89]. Stabilty of *let-7* mimics for cancer therapy have been improved [90], but delivery issues remain [91].

Therapeutic anti-miRs are currently being developed for cancer therapy, such as miR-155 for treating leukemia and lymphoma [92]. Several clinical trials of improved miRNA drug strategies, such as synthetic RNA molecules and advanced delivery technologies, are ongoing despite the failure of the first-in-human clinical trial of miRNA cancer therapy [93]. Thus far, one clinical trial in gliobastoma to assess miR-10b expression in patients with several subtypes of brain cancer is still ongoing (recruiting, NCT01849952). A miR-10b inhibitor, RGLS5579, was developed and preclinically tested for glioblastoma multiforme (GBM) [94, 86, 96, 97].

Since there is currently only one miRNA drug in clinical trials, there is little direct evidence of that miRNAs can be applied with minimal side-effects. In fact, their mechanism of action is tuning expression rather than blunting their targets which reasonably should be less detrimental to healthy tissues. In contrast to the selectivity of enzymatic protein inhibitors, miRNA drugs are developed with the idea of controlling multiple gene-components in the same or overlapping signaling-pathways. Such gene-products are not limited to proteins with enzymatic activity but could include any deregulated genes or proteins in a given disease.

## Conclusion

The role of *KRAS*, when activated through canonical mutations, has been well established in cancer. Research on *RAS*-driven cancers has focused almost exclusively on *RAS* coding mutations. However, *KRAS* without canonical mutations is still largely unexplored. *KRAS* regulation by miRNA is now emerging as a new layer of regulation [98]. Since the discovery of *let-7* miRNA, work is currently in its pre-clinical era. The control of *KRAS* expression by the *let-7* family of miRNAs is well documented. Low expression of *let-7* in human cancer correlates with high *KRAS* expression (at least in lung, breast and colorectal). The control over *KRAS* expression and activity in glioblastomas suggests that *let-7* can regulate *RAS* activity even without oncogenic mutation and that this may be a general phenomenon related to the interactions between tumour suppressor genes (*let-7*) and proto-oncogenes (*KRAS*). Open questions remain to be answered: (i) is the functional output of *KRAS* signaling essential for promoting tumorigenesis?; (ii) is the suppression of *KRAS* the dominant signalling effect in glioblastoma?; (iii) what are the general physiological effects of *let-7* miRNA dysregulation in brain tumours (if any)?; (iv) are oncogenic inhibitors currently in clinical trials for *KRAS*-driven cancers suitable for therapeutics in *KRAS* wild-type tumours? Moreover, a large fraction of miRNAs bind their targets independent of seed match complementarity at nucleotides 2–7 [99], this observation greatly affects our ability to accurately predict miRNA targets using existing algorithms. Besides the 3′UTR, miRNAs have been demonstrated to target the 5′UTR and coding sequence of mRNAs as well as other RNA species such as lncRNAs, pseudogenes, rRNAs and tRNAs [100]. We still have little work on the roles of other miRNAs and ncRNAs in brain tumors. While *let-7* shows promise as a therapeutic in brain tumors, we are left with the question of how this therapy will be implemented to help patients.

## Data Availability

No data associated in the manuscript.

## Abbreviations

miRNA: MicroRNA
*let-7*: Lethal-7
GBM: Glioblastoma
MB: Medulloblastoma
SHH: Sonic Hedgehog-activated
DIPG: Diffuse intrinsic pontine gliomas
NF1: Neurofibromin 1
PDGFR: Platelet-derived growth factor receptor
EGFR: Epidermal growth factor receptor
DMG: Diffuse midline glioma
